# Intestinal Obstruction in a 3-Year-Old Girl by *Ascaris lumbricoides* Infestation: Case Report and Review of the Literature

**DOI:** 10.1097/MD.0000000000000655

**Published:** 2015-04-24

**Authors:** Angel Medina Andrade, Yeudiel Perez, Cecilia Lopez, Stephanie Serrano Collazos, Alejandro Medina Andrade, Grecia Ortiz Ramirez, Laura Medina Andrade

**Affiliations:** From the General Surgery Department, Instituto Mexicano del Seguro Social, Hospital General Regional No. 17. Cancún, Quintana Roo, (AMA, YP, CL, SSC); Applied Biotechnique Department, Instituto Nacional de Cardiología Ignacio Chavez. Distrito Federal, México (AMA, GOR); and Internal Medicine Department, Hospital de Especialidades, Distrito Federal, México (LMA).

## Abstract

*Ascaris lumbricoides* infection affects approximately 1.5 billion people globally. Children with environmental and socio-economic risk factors are more susceptible to infestation, with serious complications such as intestinal obstruction (IO), volvulus, intussusception, and intestinal necrosis.

We present the case of a 3-year-old girl who arrived at emergency department with abdominal pain and diarrhea for the last 3 days. The previous day she took an unspecified anthelmintic. Symptoms worsened with vomiting and diarrhea, with expulsion of roundworms through mouth and anus. Physical examination revealed bloating, absence of bowel sounds, abdominal tenderness, and a palpable mass in right hemi-abdomen. Abdominal radiographs showed air-fluid levels with mild bowel distention and shadows of roundworms. The diagnosis of IO by *A lumbricoides.* infestation was established and surgical approach scheduled. During exploratory laparotomy an intraluminal bolus of roundworms from jejunum to ascendant colon was evident. An ileum enterotomy was performed and worms were removed. Fluid therapy and antibiotics for 72 hours were administered, with posterior albendazol treatment for 3 days. Patient was uneventfully discharged on the tenth day.

Reduction in parasitic load by means of improvements in sanitation, health education, and anthelmintic treatment must be implemented in endemic zones to prevent serious life-threatening complications by *A lumbricoides.* infestation, because some of them require urgent surgical treatment.

## INTRODUCTION

There are 1.5 billion people infected with *Ascaris lumbricoides.* around the world, especially in tropical and subtropical areas, representing a source of high morbidity and mortality in sub-Saharan Africa, America, China, and East Asia.^1–3^ It is a soil-transmitted helminth; its eggs hatch larvae in the duodenum and enter the circulation to the liver and lungs by sixth or eighth day. The alveolar membrane is damaged, and worms are expectorated and re-enter the intestinal tract by day eighth or tenth, where they mature into adults with subsequent oral, nasal, or anal passage. On the 24th day, they reach sexual maturity; the females produce 200,000 eggs daily and their worms grow up to 15 to 30 cm.^4^

## CASE PRESENTATION

**FIGURE 1:**
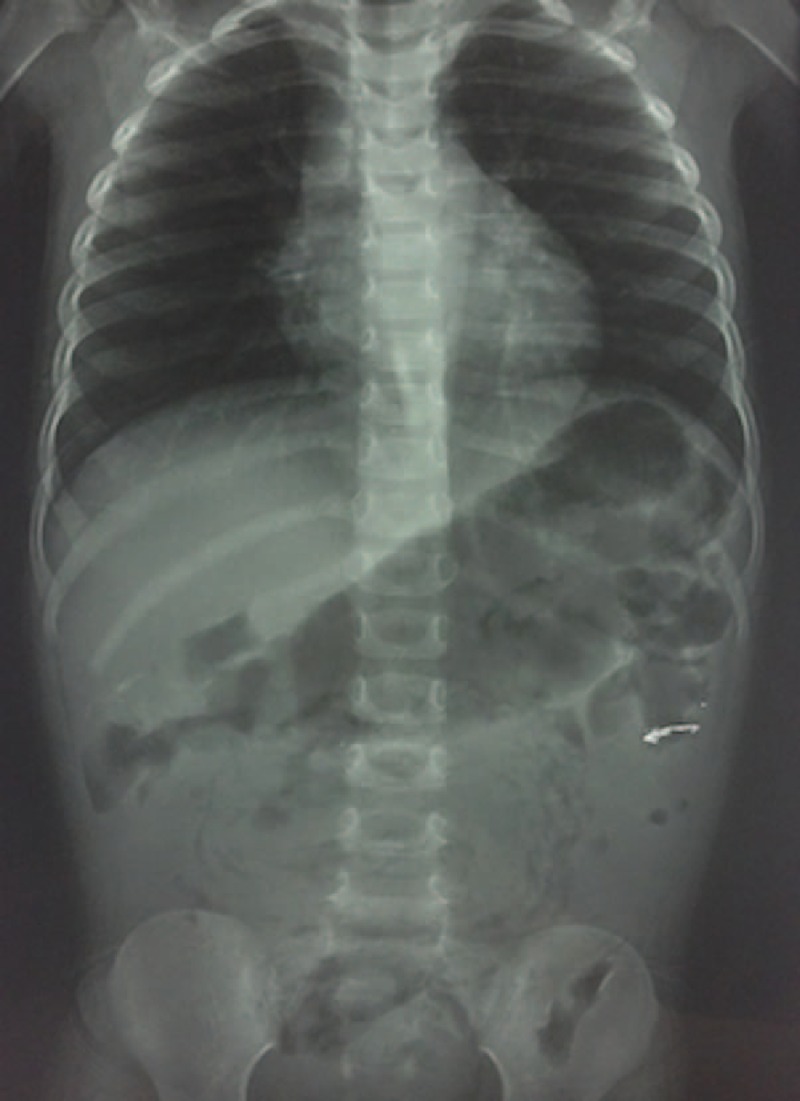
Abdominal radiography presenting multiple air-fluid levels, intestinal loops dilated, and a whirlpool image in the inferior hemiabdomen.

**FIGURE 2:**
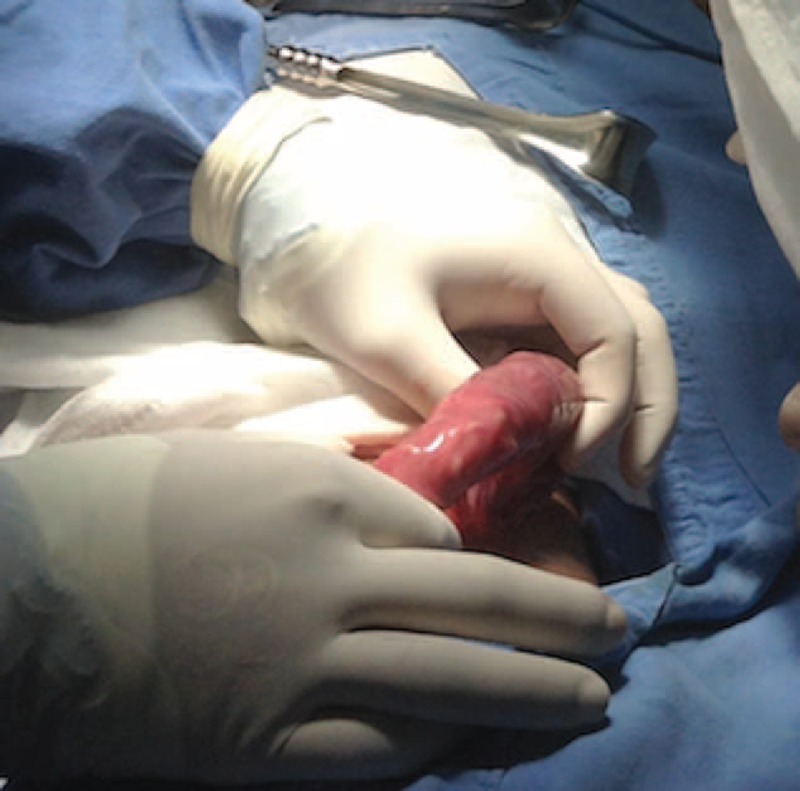
Ileum with the presence of *Ascaris* rejected to the intestinal wall.

**FIGURE 3:**
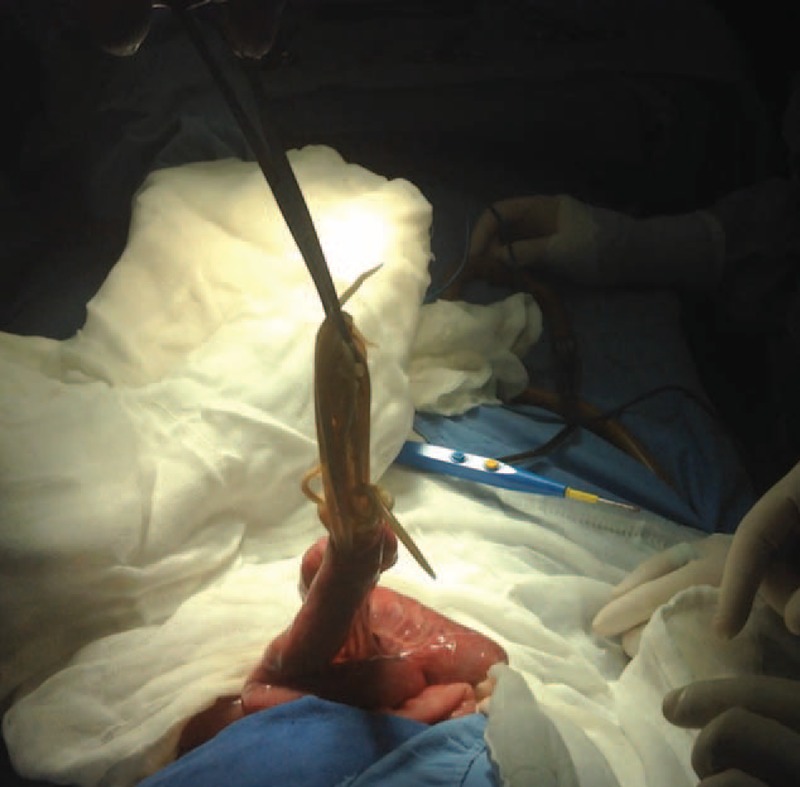
Ileum longitudinal enterotomy with the extraction of roundworms.

**FIGURE 4:**
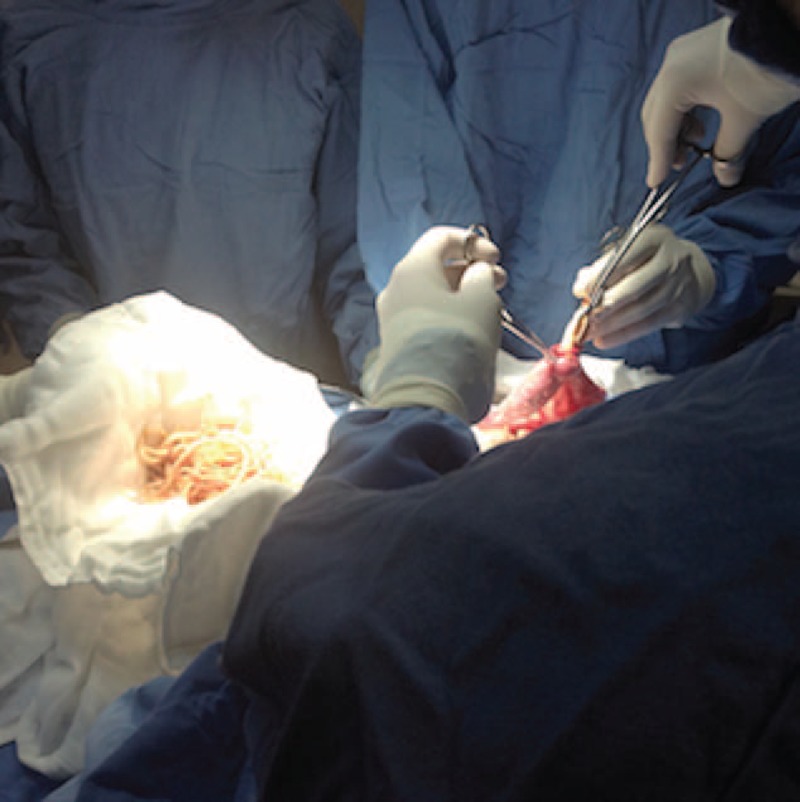
Milking roundworms from jejunum.

We present the case of a 3-year-old girl without pathological background. She is a daughter of parents with low educational and socio-economic level with no sewer service at home; her father is a farm vet. The child weighed 10 kg, measured 84 cm in height, and was in the 25 percentile weight for her age. She came down with her condition in the previous 72 hours with a diffuse abdominal pain, progressive food intolerance, vomiting, and diarrhea associated with the presence of worms in mouth and anus for the last 24 hours as referred by parents. The previous day she received an unspecified anthelmintic with increased symptoms. During the physical examination, she was irritable, dehydrated, had tachycardia, bloating, without bowel sounds, painful on palpation, and palpable serpentine-structured mass in right iliac fossa. The laboratories reported 10,300 leukocytes with eosinophilia of 10%. Abdominal radiograph showed bowel obstruction data, with multiple air-fluid levels, and a “whirlpool” image into the right iliac fossa (Figure 1). Due to intestinal obstruction (IO) and the failure of anthelmintic treatment, urgent surgical management was scheduled. During night, a pediatric surgeon is not available in the state and a general surgeon performed the surgery. Abdomen was explored finding bowel loops of jejunum, ileum, and ascending colon filled with helminths, rejected to the bowel wall (Figure 2). An ileum enterotomy was performed at 20 cm from the ileocecal valve (Figure 3), and the worms were removed with subsequent closure in 2 planes (Figure 4). Hydration and antibiotics were administered for 72 hours, and after restart of peristalsis, albendazole 100 mg twice a day for 3 days. The patient was discharged uneventfully, with a plan to deworm in 6 weeks.

## DISCUSSION

*A lumbricoides.* infection has a high prevalence worldwide, being present in almost 25% of the population. Host genetics, socio-economic status, environmental exposure, and immune response are infection determinants. After multiple rounds of chemotherapy, it has been demonstrated that individuals tend to re-acquire similar worm burdens to those harbored before treatment.^5^ Chronic infection has an important impact on growing up and development.^6^

An A lumbricoides infestation could present colecistitis, cholangitis, pancreatitis, intestinal volvulus, intussusception, appendicitis, intestinal necrosis, or IO, the latter being the more frequent.^7–9^ Complications can be observed in all ages but children between 3 and 5 years are especially susceptible, caused by large number of worms in a diminished intestinal diameter and the ileocecal valve. *A lumbricoides.* excrete a neurotoxin, which produces spasticity conducing to obstruction, and intestinal inflammation can be associated with liberation of other toxins including anaphilotoxins, hemolysins, and endocrinolysins by worms.^6,10^

Frequent symptoms are abdominal pain, nausea, vomiting, diarrhea, and presence of worms in vomit or feces. The physical examination can present abdominal tenderness, bloating, abdominal mass, or rigidity. X-rays can reveal air fluid levels and shadow of roundworms, with a “Whirlpool” image in some cases.^11^ Ultrasound will be useful in identifying thick echogenic strip with central anechoic tube; multiple linear or curvilinear echogenic strips without acoustic shadowing; “railway track” sign; “3-line” or “4-line” sign, or “bull's eye” appearances on transverse scan.^7^

In the case of intestinal subocclusion, medical treatment with intravenous fluids and electrolytes, broad-spectrum antibiotics, and nasogastric drainage must be done. Patients with complete obstruction would be candidates for laparotomy after initial resuscitation and antibiotic treatment. Single heavy dose of anthelmintic has been suggested to prevent the spontaneous resolution of the entangled bolus and may precipitate complete IO.^12^

Surgical treatment includes the extraction of the worms from the colon by enterotomy or, in case of necrosis, an intestinal resection with entero-entero anastomosis. In the presented case the extension of the infestation did not allow milked all *A lumbricoides.* to colon and could injure the intestinal wall, reason why an ileum enterotomy was done, allowing the evacuation of most roundworms and leaving the rest to medical treatment with albendazole after the restart of intestinal transit. The presence of at least 2 of the following criteria is considered for resolution: disappearance of colicky pain, beginning of defecation, or disappearance of air fluid levels. After resolution, mebendazole or albendazole 100 mg twice daily for 3 days must be started and repeated 6 weeks after discharge to eradicate any worm.^12^

Informed consent for publication of this case was signed.
